# Post-traumatic compressive pneumopericardium with spontaneous ventilation: Case report

**DOI:** 10.1016/j.rmcr.2021.101354

**Published:** 2021-01-30

**Authors:** Jamal Ouachaou, Ilyass Laaribi, Hamza Mimouni, Yassine Mellagui, Houssam Bkiyar, Brahim Housni

**Affiliations:** Intensive Care Unit, Mohammed VI University Hospital Center, Faculty of Medecine and Pharmacy of Oujda, Mohammed I University, Oujda, Morocco

**Keywords:** Pneumopericardium, Spontaneous ventilation, Blunt traumatism, Pneumothorax

## Abstract

Pneumopericardium is a rare complication of a blunt thoracic trauma. It is defined as the presence of air in the pericardial sac. There are just a few cases described in the literature.

This article brings pneumopericardium to light, reinforcing the importance of considering it within the blunt chest trauma and remarking its management with a careful monitoring for the patients whose stable or even asymptomatic with spontaneous ventilation because of the risk of tension pneumopericardium and cardiac arrest.

Diagnosis is often difficult, and it can be life-threatening by the occurrence of gas tamponade.

We report the case of a 48 years old patient victim of a severe traumatism with pneumothorax and pneumopericardium; he was stable with spontaneous ventilation.

## 1 Introduction

The Pneumopericardium is the presence of air in the pericardial sac. It is a rare condition, associated with a high mortality rate. The principal etiology is trauma; it may be seen in the context with severe blunt chest trauma, pneumothorax, pneumoperitoneum, or other causes of pneumomediastinum.

Pneumopericardium can cause serious complications such as cardiovascular collapse requiring urgent drainage.

The diagnosis is made by the computed tomography scan of the thorax and abdomen [2] that allows the additional detection of concomitant injuries. Some causes of the pneumopericardium such as tracheobronchial or esophageal tears have to be excluded by bronchoscopy. Tension pneumopericardium causing a life-threatening cardiac tamponade requires an immediate pericardial aspiration and pericardial drainage.

We report the case of a post-traumatic pneumopericardium associated with pneumothorax in a patient with spontaneous ventilation, whose evolution was favorable after thoracic drainage.

## 2 Case presentation

We present the case of a 48 years old man, a construction worker, with no significant comorbidities, who suffered a work accident by falling from a building which high estimated about 11 m. Upon arrival to emergency department, the patient was conscious, tachycardic (127 beats per minute), hyperpneic (breathing rate of 25), his blood pressure was 100/60 mmHg, and peripheral saturation of 87% which increased to 93% with supplementary oxygen physical examination found:

Clinical signs of pneumothorax (Asymmetrical lung expansion, Hyper resonant percussion), and graze in his left shoulder and a deformation of his left elbow. A FAST (Focused Assessment with Sonography for Trauma) was done and revealed a left pneumothorax.

**Fig. 1:**
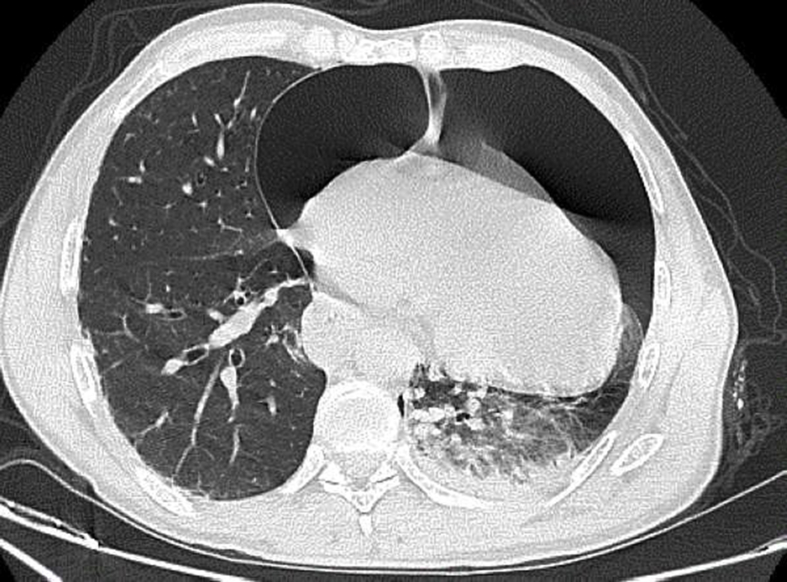
Computed tomography scan on admission, axial plane. An expressive non-tension pneumopericardium communicating with a large left pneumothorax. Left lung contusion can also be noticed.

The patient was hemodynamically stable so he had a contrast-enhanced computer tomography that showed: a large pneumothorax (more than 30% of the left hemi thorax (around 600 ml of air)), a left lung contusion, an expressive pneumopericardium (about 15mm), and fractures of lines of the left posterior arches of the 1st,10, 11, 12th ribs, fracture lines of the left middle arches of the 3rd,5th,6th ribs, a complex fracture of the left scapula and a simple fracture of the left iliopubic rami (Fig. 1), Splenic contusions, hemoperitoneum in the pouch of Douglas.

The main laboratory findings on admission were normal, as such as: Hemoglobin: 15.2g/dl,

Platelets = 153G/L, PT: 82%

The electrocardiogram showed a tachycardia, The serum troponin I level was 20 times upper normal range. Bone x-ray showed a fracture of the left elbow and the lower extremity of the left radius.

The patient's chest pain resolved within 12 hours of administration of a no steroidal anti-inflammatory agent.

After consultation with the cardiothoracic surgeons, an intercostal chest drain was indicated to relieve the pneumothorax and the pneumopericardium, so a 12-French (F) drain was inserted under local anesthesia and there was an immediate improvement in the patient's pain and all hemodynamic parameters. The drain was swinging and bubbling and he was removed on day 5. We decided not to perform any pericardial drainage procedure (pericardiocentesis or pericardial window) because we were aware that not all pneumopericardium needs draining unless tamponade effect is present.

**Fig. 2:**
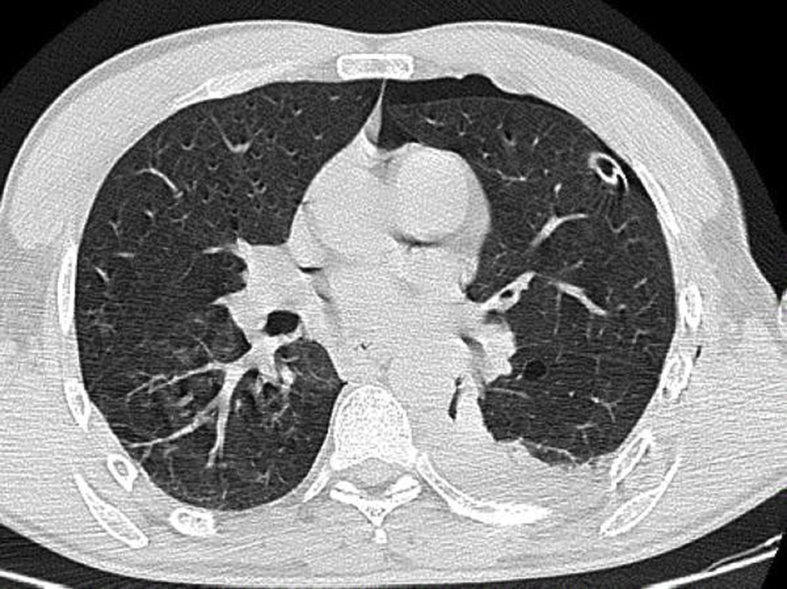
Chest CT scan showing the right-sided 12-F drain in situ and a re-expanded right lung.

Follow up chest X-ray showed a reduction in the size of pneumothorax, and A CT scan was performed 24h after, showing a regression of the left pneumothorax and complete relapse of the pneumopericardium (see Fig. 2).

The Rib fractures were assessed by the orthopedic team, which considered them stable and oriented a conservative management. However, the fracture of the elbow and radius were surgically treated. The iliopubic rami and the scapular fractures were orthopedic.

The patient was discharged home on the 8th day of his admission.

## 3 Discussion

Pneumopericardium is a rare complication of blunt or penetrating thoracic trauma and may also occur iatrogenically; Moreover, pericardial infections may also lead to pneumopericardium. Sometimes it can resolves spontaneously. nevertheless, Tension Pneumopericardium is life-threatening and requires immediate life saving intervention.

According to the literature, pneumopericardium reported during intermittent positive pressure ventilation (IPPV), especially after traumatic injuries. The mechanism of air entry into the pericardial cavity has been postulated by Macklin-what is known as “Macklin effect”- it is due to alveoli rupture which caused a sudden rise in intrathoracic pressure, leading to air leak to the pericardium via pleural cavity in the presence of a pleuropericardial tear, if the visceral pleura is disrupted causing pneumothorax, or via lung interstitium, tracking along the perivascular planes of pulmonary vessels into the mediastinum, neck, retro peritoneum and pericardium. Another mechanism would consist in direct apposition of tracheobronchial and pericardial tears.

Pneumopericardium secondary to blunt chest trauma is generally due to 1 of 3 operations:

1 penetration along pulmonary venous perivascular sheaths from ruptured alveoli to the pericardium,
2 pneumothorax with pleuropericardial tear
3) direct tracheobronchial–pericardial communication.

The 2nd mechanism is the likely cause of pneumopericardium in our patient, because he also had a large pneumothorax.

Besides cardiac tamponade is most often caused by the accumulation of blood or other fluids in the pericardial sac, it has been reported that pneumopericardium may cause cardiac tamponade. Even in patients with pneumopericardium who were initially stable status, tension pneumopericardium may develop.

Therefore, patients with asymptomatic pneumopericardium may be closely monitored to avoid escalation to cardiac tamponade.

Tension pneumopericardium leads to decreased cardiac output by inhibiting cardiac contractility and venous return to the heart; so it is a cause of hemodynamic instability. Clinically it includes hypotension, tachycardia, tachypnea, raised jugular venous pressure, muffled heart sounds, pulsus paradoxus, and classical mill wheel murmur and it can be managed by pericardial decompression either by needle pericardiocentesis or percutaneous drain placement [5]. These emergency maneuvers should only be temporary, and followed by the placement of a soft tubular drain in the operating room, by subxiphoid approach, or by open thoracotomy, or by video assisted thoracoscopic window [[3], [4]].

Few studies were conducted on post-traumatic pneumopericardium in the literature, one of the main studies spanned 60 years and compiled 32 patients with pneumopericardium after blunt chest trauma, among these 32 cases; 12 had a compressive pneumopericardium, 9 with associated pneumothorax and 3 with bronchial rupture.

Among all these patients, only two were diagnosed with spontaneous breathing and all of them were treated surgically [1].

Our patient in this case had a spontaneous breathing even with his large pneumothorax and pneumopericardum, and the FAST exam were a great help of guiding the diagnosis. The stable condition of the patient made it easier for us to perform a full-body CT scan, and only the chest drain allowed improving the patient condition. The mechanism was probably pleuropericardia communication post traumatic since it was associated with pneumothorax and the bronchoscopy showed no abnormalities.

## Ethics approval and consent to participate

Not applicable.

## Consent for publication

Not applicable.

## Availability of data and materials

Not applicable.

## Funding

Not applicable.

## Authors' contributions

Not applicable.

## Declaration of competing interest

All authors declare no conflict of interest.

## List of abbreviations

SpO2: pulsed oxygen saturation
FAST: Focused Assessment with Sonography for Traumas
